# A Singular Suicidal Attempt

**Published:** 1882-02

**Authors:** 


### A SINGULAR SUICIDAL ATTEMPT.

In April, a Parisian quarrelled with his wife about some money which he was unable to furnish. Overwhelmed by her with abuse he determined to end his days, and taking a narrow poniard, five inches in length, he placed its point against the crown of his head and with a hammer drove it into the skull to the very handle. But for all that he was no nearer to death, felt no pain and retained his faculties and unimpaired power of movement. Two physicians were now called who made traction on the handle of the instrument, but without making it budge. The patient was then taken into a neighboring work-shop, where he was held by a number of men while the dagger was withdrawn by being caught between the blades of a powerful forceps. The patient then fell to the earth, but soon regained consciousness and his natural politeness. Indeed, he conducted M. Dubrisay to his carriage bidding him adieu and "merci."

The blade of the instrument was somewhat bent near the point as though it had struck some hard body, presumably the occipital fossa. Fearing meningeal complications the patient was taken to the wards of M. Pean, whence he was dismissed after eight days without either inflammatory or paralytic symptoms.—*Journal de Med. et de Chir.*

